# Left ventricular mechanical dysfunction in obesity is exacerbated during inotropic stress cine DENSE CMR in mice

**DOI:** 10.1186/1532-429X-17-S1-Q114

**Published:** 2015-02-03

**Authors:** Christopher M Haggerty, Andrea C Mattingly, Cassi M Binkley, Sage P Kramer, Linyuan Jing, Jonathan D Suever, David Powell, Richard Charnigo, Frederick H Epstein, Brandon K Fornwalt

**Affiliations:** 1 University of Kentucky, Lexington, KY, USA; 2 University of Virginia, Charlottesville, VA, USA

## Background

Obesity is a risk factor for cardiovascular disease and mortality. Studies in both obese humans and murine models of obesity have identified changes in left ventricular (LV) mechanics (i.e., strains, strain rates, and torsion), which manifest prior to global changes in cardiac function (ejection fraction) and may represent early markers of cardiovascular disease. These data are generally acquired under resting conditions, which could mask subtle differences in the early stages of disease. We sought to evaluate LV mechanics under inotropic stress conditions with the hypothesis that mechanical deficiencies with obesity would be exacerbated under stress conditions and revealed at earlier stages of disease.

## Methods

C57BL/6J mice were randomized to either a high-fat or control diet (60%, 10% kcal from fat, respectively) for varying time intervals (n=7-10 subjects per group per time point). At each interval, LV mechanics were quantified under baseline (resting) and stress conditions (40 µg/kg/min continuous infusion of dobutamine) using cine displacement encoding with stimulated echoes (DENSE) on a 7T Bruker ClinScan. Three short-axis and 1-2 long-axis slices were acquired with 11-20 frames per cardiac cycle. Peak strain, systolic strain rates, and torsion were quantified. A linear mixed model was used to compare interactions between time and group with Benjamini-Hochberg adjustments for multiple comparisons.

## Results

**Figure 1:**
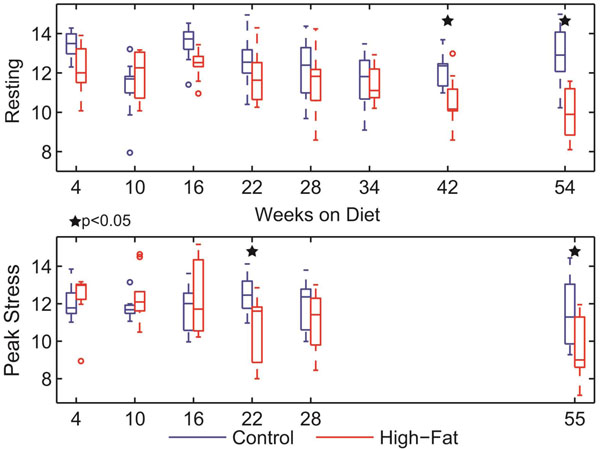
Left ventricular peak longitudinal strains at rest (top) and peak stress (bottom) at indicated time points with respect to diet. In both cases, there was an overall significant interaction of time and group, but individual time point differences (denoted by stars) were observed much earlier under stress conditions.

**Figure 2:**
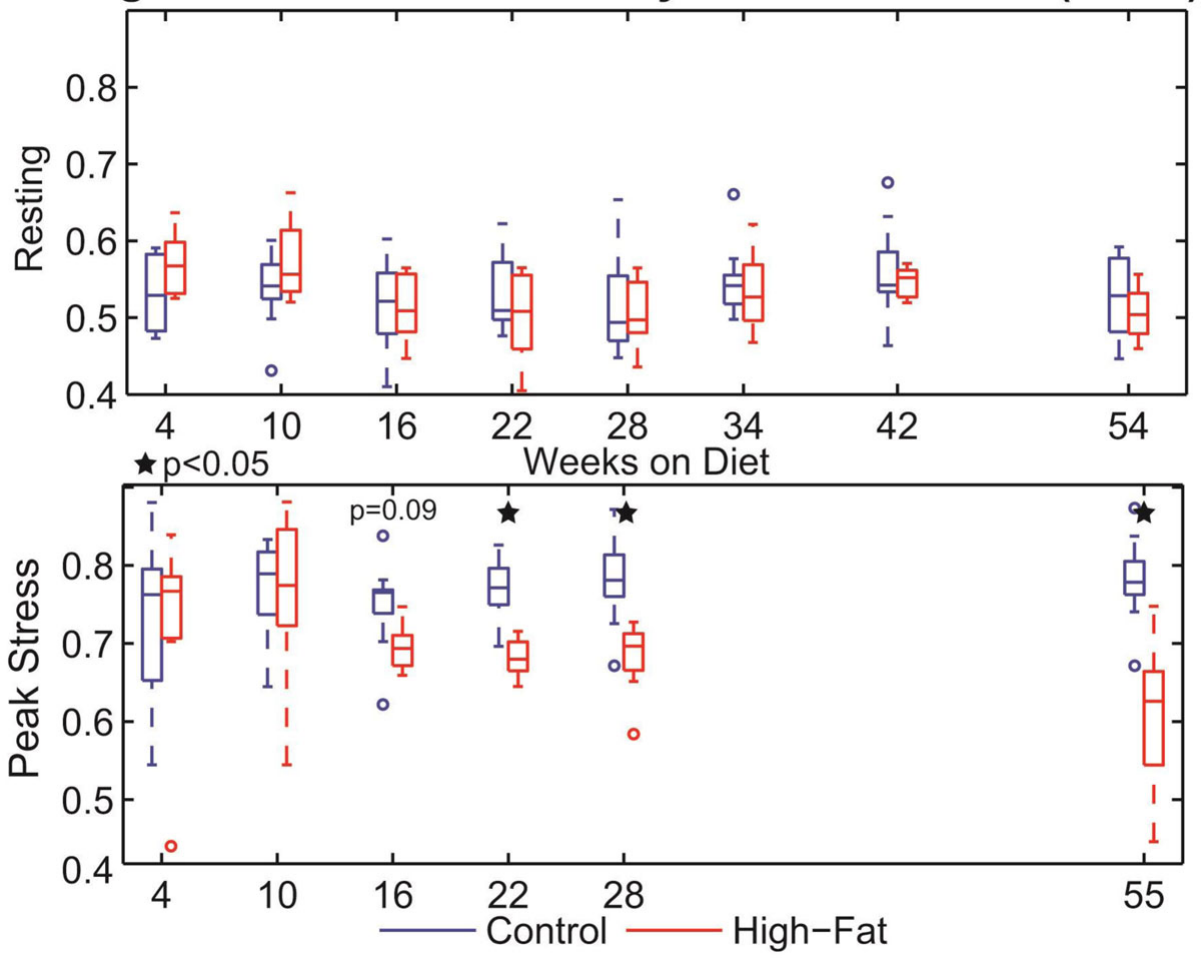
Left ventricular circumferential peak systolic strain rates at rest (top) and peak stress (bottom) at indicated time points with respect to diet. There was no significant difference between groups at rest; however, the high fat group had significantly reduced strain rate beginning at week 16 (non-significant trend) and extending through the remainder of the study (denoted by stars).

Under rest conditions, reductions in LV peak strains (Fig 1) were observed in the high-fat group after 42 weeks, with no differences in systolic strain rates (Fig 2) or torsion (not shown). Conversely, reductions in both LV peak strain and strain rates were seen under inotropic stress conditions after only 16-22 weeks on diet. Furthermore, stress CMR evaluation revealed supranormal values of LV radial strain and torsion in the high-fat group at early time points (weeks 4-10) and late deficits in peak torsion (not shown), which were not observed under rest conditions.

## Conclusions

Differences in left ventricular mechanics in obese mice are exacerbated under inotropic stress conditions. Compared to evaluation at rest, stress CMR demonstrated a broader array of mechanical dysfunction (changes in systolic strain rates and torsion in addition to peak strains) and revealed these differences at earlier time points (16-22 vs. 42 weeks). A deficit in circumferential peak systolic strain rate at peak stress was the earliest observed marker of obesity-induced ventricular dysfunction. Thus, it may be important to evaluate cardiac function in the setting of obesity under stress conditions in order to fully elucidate the presence of ventricular dysfunction.

## Funding

This work was supported by a Ruth L. Kirschstein National Research Service Award (T32 HL91812); an Institutional Development Award (IDeA) from the National Institute of General Medical Sciences of the NIH (P20 GM103527); the University of Kentucky Cardiovascular Research Center; the National Center for Research Resources and the National Center for Advancing Translational Sciences, National Institutes of Health (UL1TR000117). The content is solely the responsibility of the authors and does not necessarily represent the official views of the funding sources.

